# Flexible Quality Control for Protein Turnover Rates Using d2ome

**DOI:** 10.3390/ijms242115553

**Published:** 2023-10-25

**Authors:** Henock M. Deberneh, Rovshan G. Sadygov

**Affiliations:** Department of Biochemistry and Molecular Biology, The University of Texas Medical Branch, Galveston, TX 77555-1068, USA

**Keywords:** protein turnover, heavy water metabolic labeling, label incorporation, isotope profiles, retention time alignment

## Abstract

Bioinformatics tools are used to estimate in vivo protein turnover rates from the LC-MS data of heavy water labeled samples in high throughput. The quantification includes peak detection and integration in the LC-MS domain of complex input data of the mammalian proteome, which requires the integration of results from different experiments. The existing software tools for the estimation of turnover rate use predefined, built-in, stringent filtering criteria to select well-fitted peptides and determine turnover rates for proteins. The flexible control of filtering and quality measures will help to reduce the effects of fluctuations and interferences to the signals from target peptides while retaining an adequate number of peptides. This work describes an approach for flexible error control and filtering measures implemented in the computational tool d2ome for automating protein turnover rates. The error control measures (based on spectral properties and signal features) reduced the standard deviation and tightened the confidence intervals of the estimated turnover rates.

## 1. Introduction

Heavy water metabolic labeling followed by liquid chromatography coupled with mass spectrometry (LC-MS) is a powerful and high throughput technique for in vivo protein turnover studies [1,2,3,4,5]. The turnover rates for proteins and peptides are determined using the exponential decay modeling of the time course depletion of the monoisotopic relative isotope abundances (RIAs) obtained from the LC-MS data of heavy water labeled peptides [6,7,8,9].

Several software tools [1,8,10,11] have been developed to automate the estimation of protein turnover rate from LC-MS experiments, including d2ome [9,12]. d2ome is a powerful tool for protein turnover estimation from deuterium-labeled LC-MS experiments. The software uses nonlinear least squares regression on the time course (along with the labeling duration) of monoisotopic RIAs to determine turnover rates for proteins and peptides. The inputs for the software are the mass spectral data in the mzML [13] file format and database search results in the mzIdentML [14] format at every time point of labeling. User-specified parameters (e.g., mass accuracy) provide flexibility for the adaptation to specific experimental conditions. The outputs of the software are quantification results of turnover rates for proteins and peptides. This software has several components, including peak detection and integration, the alignment of retention time [3], isotope incorporation and label enrichment estimation [15,16], and protein turnover computation [9].

The protein turnover rate in heavy water metabolic labeling experiments is estimated as the median of the turnover rates of its constituent peptides. However, not all peptide quantifications are reliable, due to, for example, fluctuations in mass spectral intensity measurements, overlapping isotope profiles, and the co-elution of contaminants. The existing turnover rate estimation software tool, d2ome (version v1.05.5), uses a predefined built-in stringent peptide half-life filtering criteria to select well-fitted peptides and determine turnover rates for proteins. However, the predefined criteria for selecting peptides that are incorporated into the software cannot be customized by users, and at times, these criteria may not fully meet their requirements.

**Figure 1:**
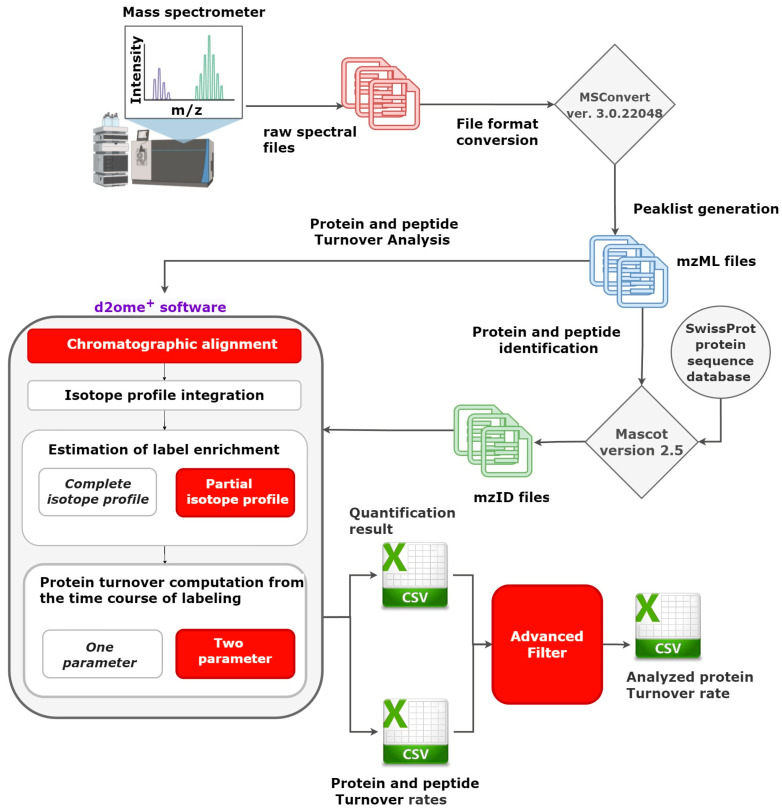
Workflow of protein turnover estimation using d2ome software. The steps indicated with the red rectangles, i.e., chromatographic alignment, the quantification of label enrichment from the partial isotope profile, two-parameter protein turnover computation, and graphical user interface (GUI) for advanced filters, are new developments in the d2ome software.

In this work, we present a bioinformatics tool for determining protein turnover rates based on user-customizable GOF measurements. The tool uses d2ome software quantification outputs to determine new protein turnover rates and their corresponding confidence intervals. The filtering parameters incorporated in this tool are the coefficient of determination (R^2^), the Pearson correlation coefficient (r), the root mean squared error (RMSE), the peptide abundance, the isotope deviation, and the number of experiments in which the peptide is identified and quantified. The tool enables users to visually inspect and validate the filtered peptides by providing the time-course plot of the experimental RIA values and their comparison with the theoretical ones. Furthermore, it generates a comma-separated output file, called Analyzed_Proteins_and_Their_Rates.csv, which contains the newly computed turnover rate, the number of peptides used in the computation, the 95% confidence interval, and the standard deviation of the protein turnover rates quantified from the LC-MS experiment. This tool is incorporated into d2ome software and is available on GitHub. Furthermore, we also report on the recent developments that are incorporated into d2ome software. The latest features include options to determine the protein turnover rates from partial isotope profiles [15], the retention time alignment [3], and the two-parameter data modeling. Figure 1 presents the overall workflow of LC-MS data processing using d2ome software. The data processing steps indicated with the red box in Figure 1 are the new features in d2ome software.

## 2. Results

### 2.1. Advanced Filters to Facilitate Protein Turnover Rate Analysis

In heavy water metabolic labeling experiments, the incorporation of ^2^H atoms into amino acids during labeling remodels the distribution of mass isotopomers. These changes were recorded in the LC-MS profiles of peptides and used to determine turnover rates for proteins and peptides. Exponential decay functions were used to model the time-course depletion of the monoisotopic RIA [17,18], Equation (1):

(1) $$I_{10}\left(t\right)={\;I}_{0}^{\mathrm{asymp}}+\left(I_{0}\left(0\right)-{\;I}_{0}^{\mathrm{asymp}}\right)e^{-\mathrm{kt}}\;$$

(2) $$I_{0}\left(t\right)=\frac{A_{0}\left(t\right)}{\sum_{i=0}^{5}A_{i}\left(t\right)}$$

where I_0_(0) is the monoisotopic RIA of the unlabeled (natural) peptide determined as the normalized abundance of the monoisotope from the complete isotope profile of a peptide [16,19] (Equation (2)); I_0_^asymp^ is the monoisotopic RIA at the plateau of labeling; t is the labeling duration; and k is the turnover rate. A_i_(t) is the raw abundance of the i^th^ mass isotopomer at the labeling time point t.

d2ome software utilizes Equation (1) to determine the turnover rates for peptides from time-course LC-MS data of heavy water labeled samples. The accuracy of the peptide’s turnover rate is evaluated by comparing the experimental monoisotopic RIAs quantified from the LC-MS data with the theoretical values computed for each labeling duration using Equation (1). The coefficient of determination (R^2^), Pearson correlation coefficient (r), and RMSE values are used to measure GOF.

The estimated turnover rate for peptides of the same protein may vary due to fluctuations in mass spectral intensity measurements and overlapping isotope profiles caused by co-eluting contaminants in complex proteome mixtures. For the accurate determination of protein turnover rates, peptides are filtered based on their GOF characteristics. To be eligible for the estimation of protein turnover rate, peptides must meet one of two filtering criteria based on their rate constant values. If the peptide has a rate constant of less than 0.01 day^−1^ (k < 0.01 day^−1^) (slow turnover proteins), it must satisfy the threshold of RMSE < 0.01. Otherwise (k ≥ 0.01 day^−1^), it must have R^2^ > 0.9, r > 0.9, and RMSE < 0.05. To exclude any aberration from the peptides that passed the GOF threshold, Grubbs’ outlier-detection algorithm [20] is applied. The protein turnover rate is computed as the median of the turnover rates of peptides that meet the filtering criteria. For the majority of the peptides, the second filtering criteria is used. However, for the peptides with slow turnover, the R^2^ value is not a good quality measure due to the small differences in monoisotopic RIAs in labeled and unlabeled samples.

Originally, in d2ome, the thresholds described above were used as GOF cutoff measures for peptides to achieve robust protein turnover estimation using a heuristic approach. These values are not user-customizable and, on some occasions, may come short of satisfying a user’s specific expectations. Thus, we developed a GUI application to enable users to input the GOF characters that fit their expected criteria. The user-customizable GOF parameters incorporated in the software include RMSE, R^2^, r, standard deviation (SD), the number of experiments that the peptide is identified in, isotope deviation, and the abundance of the peptide.

The input for the advanced filtering tool is the rate-constant-quantification outputs from the d2ome software, called “ProteinName.RateConst.csv”. These files are generated for each identified protein in the LC-MS dataset and contain detailed information about the computed peptides’ turnover rate and the corresponding GOF characteristics. Each file contains the peptide’s sequence, its charge and rate constant, and the corresponding lower and upper bounds of the confidence interval (CI), the GOF measures between theoretical fit and experimental RIAs, the absolute deviation between the theoretical and experimental isotope profiles of the unlabeled peptide, the sequence mass-to-charge ratio (*m*/*z*), the number of accessible hydrogens (N_EH_), the number of data points (NDP), and the average abundance of the monoisotope. The GOF measures included in the “ProteinName.RateConst.csv” file are the Pearson correlation coefficient (r), the coefficient of determination (R^2^), the root mean squared error (RMSE), and the standard deviation (SD).

The software uses the GOF thresholds set by the users to filter peptides and compute a new turnover rate and 95% CI for proteins. The output of the software is a comprehensive new “Analyzed_Proteins_and_Their_Rates.csv” file that contains the newly computed turnover rates of proteins and their corresponding CIs. In accordance with the user GOF parameter, the filters lower the standard deviation of the turnover rate and result in a tighter CI.

**Figure 2:**
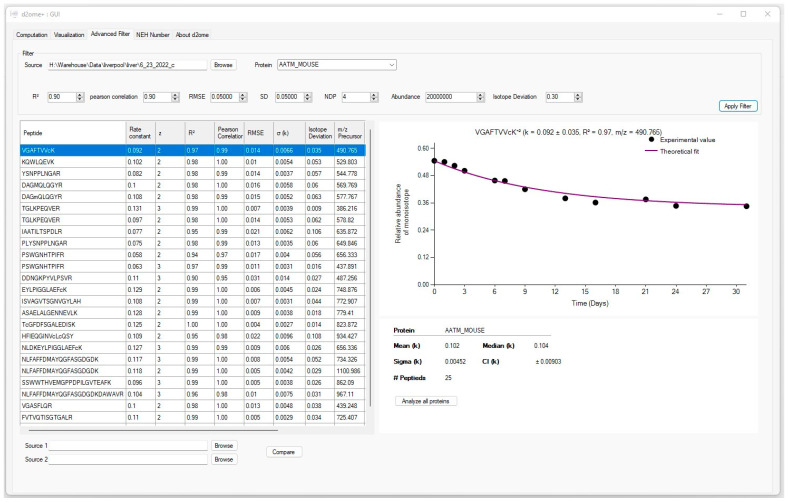
The graphical user interface (GUI) for advanced tool for protein peptide filtering. User-defined stringent filtering parameters can result in robust protein turnover estimation and improve the confidence interval for protein turnover estimations.

Figure 2 presents a sample screenshot of the advanced peptide-filtering tool. The window has three main sections. The first section, located at the top of the window, contains the input controls to enter the GOF thresholds. The left side of the window shows protein peptides and their corresponding turnover rates with GOF measures in a tabular format. The right side of the window presents a graphical visualization for the comparison of the time-course monoisotopic RIAs estimated from the isotope profiles with the theoretical fit.

The performance of the filtering tool was evaluated using a benchmark dataset acquired from a recent work that reported a large-scale LC-MS murine liver proteome study [21]. The dataset contains raw mass spectral data, database search results, and quantification outputs that were obtained from eighteen C57/BL6J male mice liver tissues using an Orbitrap Eclipse mass spectrometer at nine different labeling durations (0,1, 2, 3, 4, 5, 6, 14, and 21 days). The experiments were described in detail in the original publication. In brief, for day zero (unlabeled samples), two mice were randomly selected and were used to estimate the natural isotope abundances. The remaining mice were IP-injected with 750–960 ul of 99.9% D_2_O that was made isotonic with 0.9 g NaCl *w*/*v*. They were immediately given free access to 8% enriched (*v*/*v*) deuterated water for variable labeling durations [15,21]. At each labeling duration, two randomly chosen mice were sacrificed, and dissected livers were used to prepare the samples for LC-MS analysis. ThermoFisher Eclipse Orbitrap mass spectrometer was operated using data-dependent acquisition (DDA) to obtain the raw mass spectral data from the liver samples. Proteowizard MSConvert tool [22] was used to convert the raw mass spectral data to mzML format, and the Mascot database search engine [23] was used to identify peptides from tandem mass spectra data. The turnover rates of proteins and peptides were determined from the spectral data and the database search results using d2ome software.

**Figure 3:**
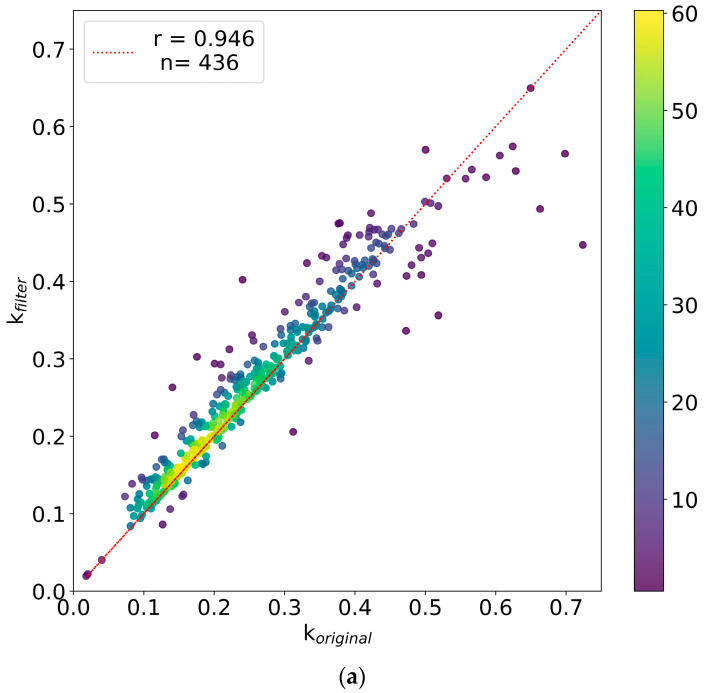

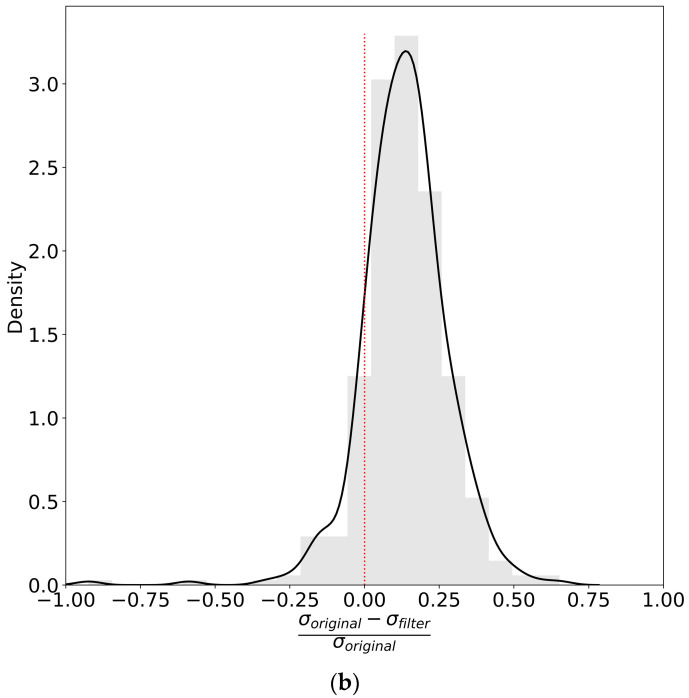
The advanced filtering technique improves the standard deviation and 95% confidence intervals of computed turnover rates: (**a**) scatter plot and heat map of protein turnover rates before (*x*-axis) and after (*y*-axis) using advanced filtering tool, (**b**) distribution of the relative differences between the standard deviation of the original d2ome output and the newly computed value using the advanced filtering tool.

Figure 3a,b present the comparison of the computed turnover rates and their corresponding standard deviations before and after using the advanced filtering tool. R^2^ ≥ 0.95, r ≥ 0.95, RMSE ≤ 0.05, SD ≤ 0.05, NDP ≥ 4, abundance ≥ 2 × 10^7^, and isotope deviation ≤ 0.3 thresholds were used to recompute the protein turnover rates and their corresponding 95% confidence intervals. For the comparative analysis, 436 proteins with at least five peptides that satisfied the filtering criteria were selected. Figure 3a shows a scatter plot and heat map of protein turnover rates before (*x*-axis) and after (*y*-axis) using the filtering thresholds. The correlation coefficient between the original d2ome output (k_original_) and the newly computed turnover rates using the advanced filtering (k_filter_) was 0.94. For 80% of the proteins, the relative difference between the original and newly computed turnover rates is less than 15%. Overall, the observed change in turnover rates due to the filtering criteria was small. However, the change in the standard deviation and the confidence intervals of protein turnover rates is significant. Figure 3b shows the distribution of the relative differences between the standard deviations of the original d2ome outputs and the newly computed values using the advanced filtering tool. The filtering technique improves the standard deviations and the confidence intervals of the computed turnover rates for 88% of proteins. In addition, the standard deviation was improved by more than 15% and 25% for 45% and 17% of the proteins, respectively. The standard deviation for protein turnover rate is computed as the harmonic mean of the standard deviation of its constituting peptides. Consequently, the improvements in the standard deviation result in a tighter confidence interval.

### 2.2. Recent Developments in d2ome Software

#### 2.2.1. Quantification of Label Enrichment from Partial Isotope Profiles

The incorporation of deuterium into amino acids results in a composite profile that contains both labeled and unlabeled versions of the peptide. The existing methods for estimating the label incorporation use the complete isotope profiles of a peptide to determine the normalized monoisotopic RIA [1,24]. This technique has an advantage in averaging out measurement errors that arise due to the limitations in spectral accuracy. However, this technique fails to compute the accurate monoisotopic RIA when the isotope profile is distorted. Due to the complexity of the mammalian proteome, it is common for target peptides to co-elute with contaminants and result in overlapping and distorted isotope profiles. The traditional approach, which uses the first six heavy mass isotopomers to determine RIA, results in inaccurate estimations of label incorporation. It has been observed that more than half of the peptides quantified using this technique exhibit low GOF characteristics (with an R^2^ value less than 0.8) and cannot be utilized in determining the protein turnover rate [17,24].

To address this problem, we have introduced a new algorithm to estimate label incorporation for a peptide from the ratio of any pair of its mass isotopomers [15,25]. This algorithm only uses the ratio of raw abundances from two unaffected mass isotopomers to determine the monoisotopic RIA in overlapping peptide isotope profiles. This technique has doubled the number of high-quality quantified peptides (R^2^ ≥ 0.95) and improved the CIs of the computed turnover rates.

**Figure 4:**
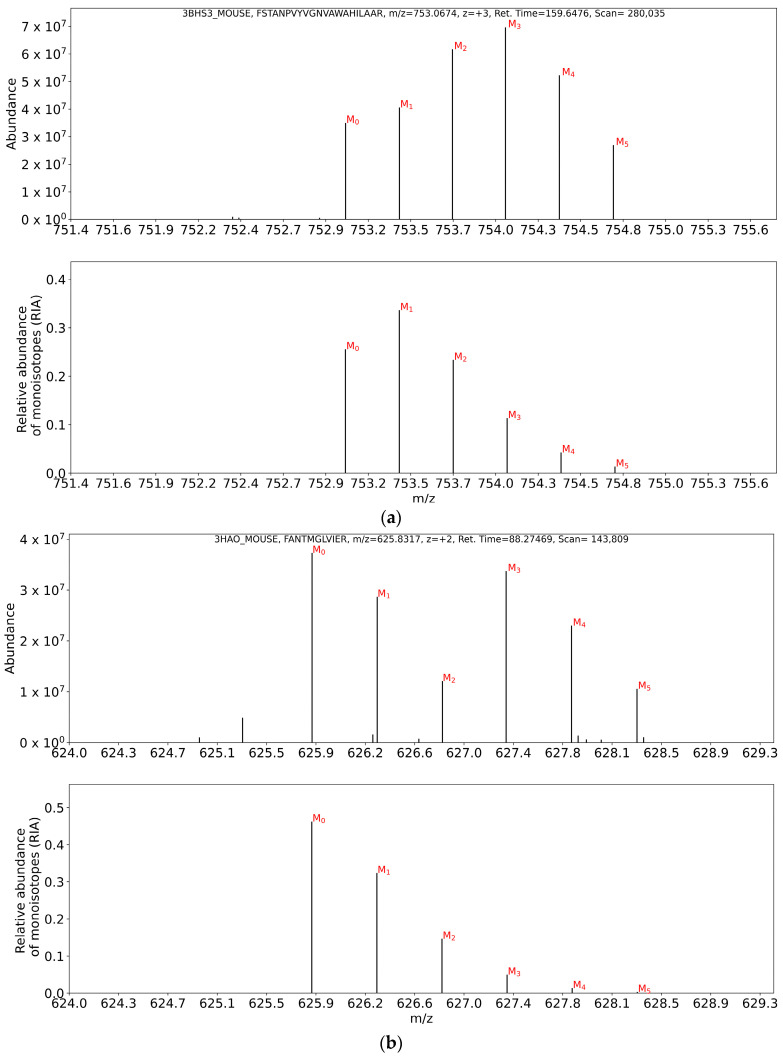
Monoisotopic RIAs can be accurately determined from overlapping isotope profiles by using the ratios from two mass isotopomers. (**a**) Experimental isotope profile from the unlabeled sample for the FSTANPVYVGNVAWAHILAAR^+3^ peptide from 3BHS3_MOUSE protein (**top**) in comparison with the theoretical isotope profile (**bottom**); (**b**) overlapping isotope profile from the unlabeled sample of FANTMGLVIER^+2^ (3HAO_MOUSE).

Figure 4 presents the common type of isotope profile overlap (top plot) in comparison with the theoretical spectrum from unlabeled samples (bottom plot). Figure 4a shows the interference in the isotope profile for the FSTANPVYVGNVAWAHILAAR^+3^ peptide of the 3BHS3_MOUSE protein. As seen from the figure, the M_2_–M_5_ mass isotopomers were affected by the interferences from a co-eluting contaminant. Therefore, the complete isotope profile underestimates the monoisotopic RIA. However, the estimation of the label enrichment from the unaffected M_0_ and M_1_ can be used to accurately determine I_0_(t). Similarly, Figure 4b shows a distorted isotope profile of the FANTMGLVIER^+2^ peptide from 3HAO_MOUSE. The interference affects the intensity of M_3_–M_5_ mass isotopomers. The estimation of label enrichment from the partial isotope profile can be used to accurately compute the monoisotopic RIA from the unaffected M_0_, M_1_, and M_2_ mass isotopomers.

**Figure 5:**
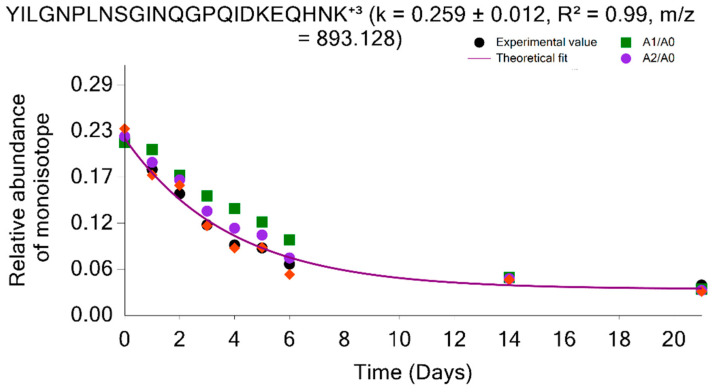
Label incorporation estimation using two mass isotopomers reproduces the monoisotopic RIA determined using the complete isotope profile. The solid magenta line indicates the theoretical fit from the computed turnover rate. The black circle, green rectangle, magenta circle, and orange diamond represent the monoisotopic RIAs determined using the complete isotope profile, A_1_(t)/A_0_(t), A_2_(t)/A_0_(t) and A_2_(t)/A_1_(t), respectively.

**Figure 6:**
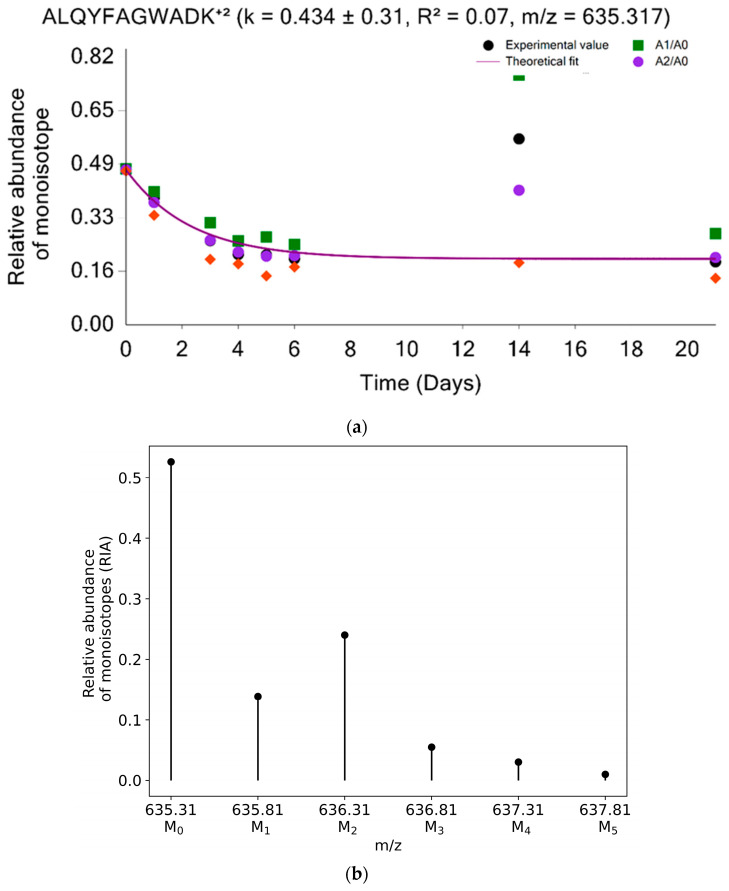
Estimation of label enrichment using partial isotope profile can be used to accurately determine monoisotopic RIA from overlapping isotope profiles. (**a**) The black circle, green rectangle, magenta circle, and orange diamond all show the monoisotopic RIAs, which were computed using the complete isotope profile, A_1_(t)/A_0_(t), A_2_(t)/A_0_(t), and A_2_(t)/A_1_(t). The theoretical fit from the computed turnover rate is shown by a solid magenta line. (**b**) Isotope profile of ALQYFAGWADK^+2^ (AL1A7_MOUSE) peptide at 14 days of heavy water metabolic labeling.

Figure 5 and Figure 6 depict the time-course comparison of the experimental and theoretical monoisotopic RIA determined from complete and partial isotope profiles for two peptides (YILGNPLNSGINQGPQIDKEQHNK⁺³ and ALQYFAGWADK^+2^) of the AL1A7_MOUSE protein. Without co-elution, both methods reproduce similar RIA values, as shown in Figure 5. Figure 6a demonstrates the sample-improvement data point using the RIA values from the A_2_(t)/A_0_(t) from the distorted isotope profile. Figure 6b presents the peptide isotope profile at 14 days of deuterium labeling. As shown in Figure 6a, the experimental RIA computed from the six mass isotopomers for 14 days of labeling duration is overestimated due to the co-elution of the peptide with a contaminant that resulted in distorted isotope profiles. As a result, the computed GOF measures for the peptide are too low to be used for protein turnover estimation (R^2^ = 0.07 and RMSE 0.123). Using the RIA values determined from A_2_(t)/A_0_(t), the peptides GOF can be improved from 0.07 R^2^ value to 0.99 with an RMSE value of 0.01. This will make the peptide usable for protein turnover estimation. Similarly, the RIA values from the other ratios (A_1_(t)/A_0_(t), and A_2_(t)/A_1_(t)) are also applicable to improve the peptide’s goodness-of-fit measures depending on the degree of the isotope profile overlap. Comprehensive statistics about the performance of the two-mass isotopomers method and its comparison with the complete isotope profile have been presented elsewhere [15].

#### 2.2.2. Retention Time Alignment

At each labeling duration, peptides are detected and quantified using their tandem mass spectra and precursor m/z. However, due to the stochastic nature of the data-dependent acquisition (DDA) techniques, a significant number of “missing values” are observed across experimental datasets. The “missing values” problem becomes more prominent in the metabolic labeling of heavy water. The incorporation of ^2^H atoms into amino acids increases the abundance of heavy-mass isotopomers. As a result, the isotope distribution of the fragment ions is different from those of natural peptides. These differences affect the performance of conventional database search engines and reduce the number of confidently identified peptide spectrum matches.

Match between runs (MBR) and accurate mass and chromatographic time alignment techniques have been widely used to address the “missing value” problem [26,27,28]. However, this technique does not account for time series samples from metabolic deuterium-labeling experiments. This is due to the significant changes in isotope profiles of peptides caused by the incorporation deuterium, as well as the retention time shifts in chromatograms obtained at different labeling durations. To address this issue, we implemented an algorithm based on correlation-optimized time warping to align peptide retention time between heavy-water-labeled LC-MS experiments [3].

**Figure 7:**
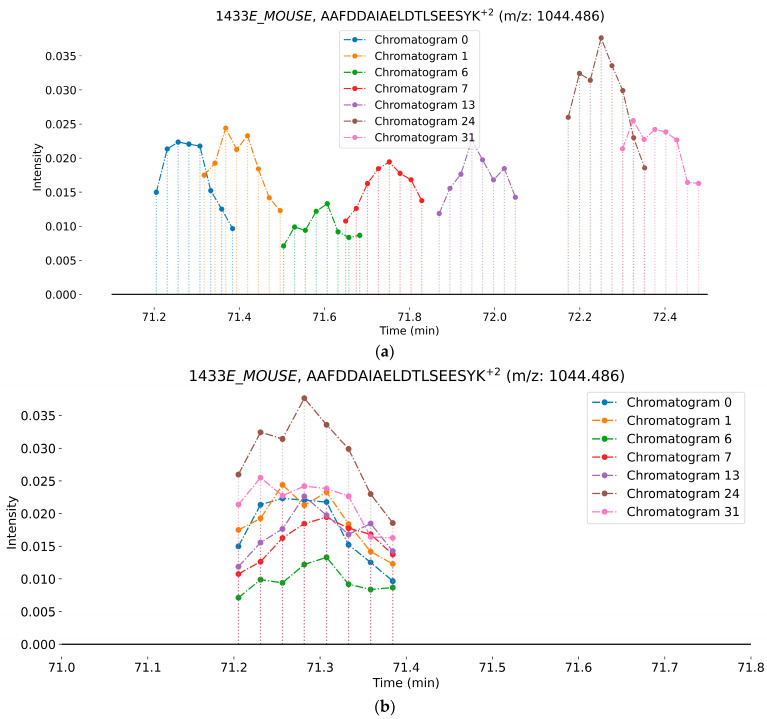
The overlay of the elution windows for the AAFDDAIAELDTLSEESYK^+2^ peptide at six labeling durations: (**a**) before retention time alignment and (**b**) after retention time alignment. The vertical dotted lines and the circles at the top of each line indicate the intensity of the base peak at the corresponding retention time. The line connecting the circle indicates the elution window of the peptide at a specific labeling duration. The different colors represent the labeling duration for each chromatogram.

Figure 6a,b present the elution profile of the AAFDDAIAELDTLSEESYK^+2^ peptide of the 1433E_MOUSE protein acquired from LC-MS chromatograms at seven different labeling durations (0, 1, 6, 7, 13, 13, 24, and 31 days). Figure 7a shows the retention time shift of the peptides in different experiments. The maximum shift observed for this peptide is 65.7 s between the chromatograms acquired from the unlabeled sample and 31 days labeled sample. After applying the technique for retention time alignment, we were able to align the most significant peaks of the chromatograms in the elution window of the peptide, as shown in Figure 7b.

**Figure 8:**
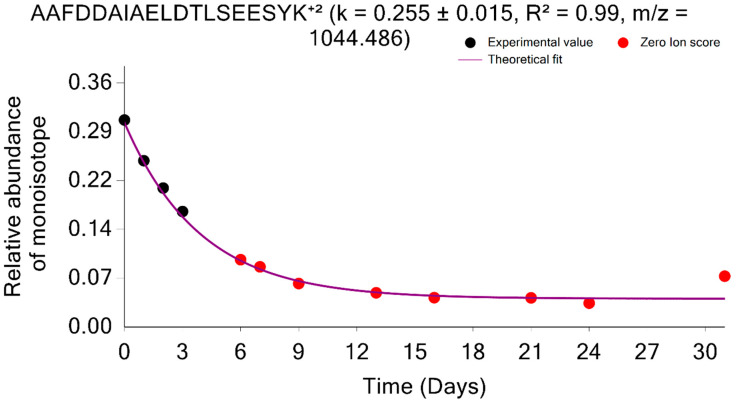
Time course plot of monoisotopic RIAs for the AAFDDAIAELDTLSEESYK^+2^ peptide from the 1433E_MOUSE protein. The experimental time points quantified using the match between runs are shown in red.

The retention time alignment followed by MBR is implemented in d2ome software as a solution to mitigate the “missing value” problem. MBR is used to transfer peptide features from one experiment where the peptide is fragmented and identified to the experiment where it was not identified. Prior to the transfer, the experimental RTs are aligned to minimize the risk of incorrect transfers. This technique has been thoroughly validated in different data sets and has consistently increased the number of quantified peptides for quantitative analysis. In addition, this algorithm improves the CI and the SD of the estimated turnover rates by increasing the number of experiments in which the peptide is quantified. For instance, the peptide used in Figure 7, AAFDDAIAELDTLSEESYK^+2^, was not identified in the experiments with labeling durations of 6, 7, 9, 13, 16, 21, 24, and 31 days. However, by using RT alignment and the MBR technique, we were able to accurately quantify the peptide in experiments where it was undetected. In Figure 8, the red dots represent the quantified points obtained using RT, followed by the MBR technique.

#### 2.2.3. Two-Parameter Modeling

d2ome utilizes a nonlinear regression model to estimate the turnover rates of peptides. This model estimates the fitting parameters by minimizing the sum of squared errors between the experimental data points and the theoretical values that are computed using Equation (1). The optimization technique implemented in the software is the Broyden–Fletcher–Goldfarb–Shanno algorithm (BFGS) [9,29]. This algorithm takes the labeling durations, the time course experimental monoisotopic RIA values, and the normalized theoretical monoisotopic RIA at the plateau of labeling and fits those values to Equation (1) and determines the only parameter, which is the turnover rate (k). Hence, it is referred to as one-parameter data modeling. This data-modeling technique has been the default method for protein turnover estimation in d2ome software.

In this work, we introduce the incorporation of the two-parameter data modeling approach in d2ome software. This approach uses the same equations as one parameter data modeling technique, Equation (1), to model the experimental data points. Unlike the one-parameter model, this approach fits two parameters: the turnover rate and the monoisotopic RIA at the plateau of labeling. In one-parameter modeling, the RIA at the plateau of labeling was determined using Equation (3) shown below:

(3) $$I_{0}^{\mathrm{asymp}}={\;I}_{0}\left(0\right){\left(1-\frac{p_{W}}{1-p_{H}}\right)}^{N_{\mathrm{EH}}}$$

where $I_{0}\left(0\right)$ is the monoisotopic RIA of the unlabeled peptide and p_W_ is the body water enrichment in deuterium. Here, we determine I_0_^asymp^ by fitting the experimental points to the data model mentioned above. The optimization algorithm used in this method is also BFGS. To ensure the accurate estimation of parameters, the optimization parameters for the BFGS algorithm are set accordingly. This includes the number of iterations and the minimum absolute error difference between two successive iterations.

The optimization algorithm searches for the best-fit values for the parameters from unrestricted search space. However, the parameters used in the model have a range limit. Thus, the turnover rate values for peptides cannot be negative, and the asymptotic normalized RIA values cannot be greater than the natural RIA. To account for these restrictions, d2ome incorporated parameter transformations as shown in Equations (4) and (5):

(4) $$k=e^{-\theta }$$

(5) $$I_{0}^{\mathrm{asymp}}=\frac{I_{0}\left(0\right)}{\left(1+e^{-\alpha }\right)}$$

θ and α are unconstrained parameters. Overall, the two-parameter approach enables users to determine the turnover rates and the asymptotic RIA values for peptides simultaneously. This will give additional options to evaluate the accuracy of the estimated turnover rate and GOF by comparing the asymptotic monoisotopic RIA quantified from the experimental LC-MS data with the fit parameter obtained from the two-parameter model.

## 3. Conclusions

In this work, we described a user-customizable tool for the estimation of the protein turnover rate. This tool enables users to utilize their standard GOF measures to compute protein turnover rates instead of d2ome’s built-in stringent criteria. The output from the software is a comprehensive summary file that contains proteins identified in the LC-MS dataset and their turnover rates with corresponding CIs. The tool is incorporated into d2ome software and available on GitHub at https://github.com/rgsadygov/d2ome (accessed on 20 September 2023).

This work also summarizes the latest advancements in d2ome software. The recent developments include two-parameter data modeling for protein turnover estimation, retention time alignment to address the “missing value” problems in deuterium metabolically labeled experiments, and the estimation of label incorporation from partial isotope profiles to resolve the complexity of the mammalian proteome. These methods increase the proteome coverage and number of quantified peptides, reduce the SDs of the turnover rates, and improve their CIs.

## Data Availability

Tools reported in this paper are available in the GitHub repository: https://github.com/rgsadygov/d2ome (accessed on 20 September 2023).

## Abbreviations

CI—confidence interval; GOF—goodness of fit; LC-MS—liquid chromatography and mass spectrometry; m/z—mass-to-charge ratio; NDP—the number of data points; NEH—the number of hydrogen sites accessible to deuterium in heavy water; RIA—relative isotope abundance; RMSE—root mean squared error; SD—standard deviation.
